# Identification and Full-Genome Characterisation of Genomoviruses in Cassava Leaves Infected with Cassava Mosaic Disease

**DOI:** 10.3390/v17111418

**Published:** 2025-10-25

**Authors:** Olabode Onile-ere, Oluwagboadurami John, Oreoluwa Sonowo, Pakyendou Estel Name, Ezechiel Bionimian Tibiri, Fidèle Tiendrébéogo, Justin Pita, Solomon Oranusi, Angela O. Eni

**Affiliations:** 1 Department of Biological Sciences, Covenant University, Km 10 Idiroko Road, Ota 12212, Ogun State, Nigeria; 2 Central and West African Virus Epidemiology Program, Covenant University Hub, Km 10 Idiroko Road, Canaan Land, Ota 12212, Ogun State, Nigeria; 3 Laboratory of Virology and Plant Biotechnology, Institute for the Environment and Agricultural Research (INERA), Ouagadougou 01 BP 476, Burkina Faso; 4 WAVE Regional Center of Excellence for Transboundary Plant Pathogens, Université Felix Houphouët-Boigny (UFHB), Abidjan 01 BPV 34, Côte d’Ivoire

**Keywords:** Genomovirius, CRESS-DNA virus, viromics

## Abstract

This study identified and characterised three Genomoviruses during a circular DNA-enriched sequencing project aimed at assessing the evolution of Cassava mosaic begomoviruses in Nigeria. Using a combination of rolling circle amplification, Oxford Nanopore Sequencing and targeted amplicon sequencing, three full-length Genomovirus genomes were recovered. The recovered genomes ranged from 2090 to 2188 nucleotides in length, contained two open reading frames (Rep and CP) in an ambisense orientation and shared between 84.81 and 95.37% nucleotide similarity with isolates in the NCBI GenBank repository. Motif analyses confirmed the presence of conserved rolling circle replication (RCR) and helicase motifs in all three isolates; however, one isolate lacked the RCR II motif. Phylogenetic inference using Rep and CP nucleotide sequences suggested that the isolates belonged to a divergent lineage within the Genomovirus family. These findings expand current knowledge of Genomovirus diversity and highlight the potential of cassava as a source for identifying novel CRESS-DNA viruses.

## 1. Introduction

Genomoviruses (family *Genomoviridae*) are a rapidly expanding group of single-stranded DNA viruses characterised by circular genomes of between 1.8 and 2.4 kb and encoding two major proteins, namely, rolling circle replication initiator protein (Rep) and a capsid protein (CP) in an ambisense orientation [1]. The Genomovirus Rep protein shows significant homology to those found among the Geminiviruses; however, the CP is highly divergent and does not show any significant similarity to CPs from other viral families.

Following the establishment of the *Genomoviridae* family in 2016 [1,2], the number of sequenced genomes has increased significantly, resulting in the expansion of the family from one to ten genera and over 230 species according to the International Committee on Taxonomy of Viruses (ICTV) reports [1]. While Genomoviruses have been isolated across a wide variety of hosts and environments, the true host range remains largely unknown as they are typically recovered from metagenomic sequencing of environmental, plant or animal samples. To date, *Sclerotinia sclerotiorum* hypovirulence-associated DNA virus 1 (SsHADV-1) which infects the phytopathogenic fungus *S. sclerotiorum* [3] and the *Fusarium graminearum* gemytripvirus 1 which infects crop fungus *F. graminearum* [4] are the only two Genomoviruses that have been definitively linked to a host.

Despite their widespread prevalence, very little is known about the biology and ecological roles of Genomoviruses [1]. They are not known to cause any disease in animals or plants and are typically regarded as part of the cryptic eukaryotic virome. In this study we employed a combination of rolling circle amplification, Oxford Nanopore Sequencing and amplicon sequencing to retrieve the full genomes of Genomoviruses from Cassava mosaic disease (CMD)-infected leaves.

## 2. Materials and Methods

### 2.1. Sample Collection

Cassava leaf samples infected with Cassava mosaic disease (CMD) were collected during routine surveys conducted across Nigeria between 2015 and 2022 by the WAVE program and stored in Herbarium presses [5,6]. As part of routine surveillance efforts to understand the diversity of geminiviruses causing CMD symptoms, a subset of 91 cassava leaf samples, collected from across 33 states of Nigeria, were randomly selected for DNA sequencing. Total DNA was extracted following the CTAB extraction procedure as previously described [7]. Exactly 100 mg of the dried sample was ground in 1.5 mL of CTAB buffer (2% CTAB, 100 mM Tris, 20 mM EDTA, 1.4 M NaCl and 1% PVP), incubated at 60 °C for 1 h and then spun at 20,000× *g* for 10 min to remove cellular debris. The resulting supernatant was combined with an equal volume of chloroform–isoamyl alcohol (24:1) and then spun at 20,000× *g* for 10 min. This step was repeated a second time after which the resulting supernatant was incubated with iso-propanol for 30 min at −20 °C, followed by centrifugation at 20,000× *g* for 10 min. The pellet was washed in ice-cold 70% ethanol for 20 min to precipitate the DNA before a final spin at 20,000× *g*. The pellet was allowed to dry before dissolving in 20 µL of Tris-EDTA buffer (10 mM Tris–HCl [pH 8.0] + 1 mM EDTA). The circular DNA in the samples were enriched by rolling circle amplification (RCA) using the Illustra Templiphi Kit (GE Healthcare, IL, USA) following the manufacturer’s instructions.

### 2.2. Initial Sequencing, De Novo Assembly and Taxonomic Classification

Sequencing libraries were prepared from RCA amplicons using the ONT native barcoding Kit 96 V14 (ONT, OX, UK) [8] and pooled following the ligation sequencing protocol. The libraries were then sequenced using the R10.4.1 flow cell on the MinION sequencer (ONT, OX UK). After sequencing, raw reads were basecalled using the Dorado basecaller version 0.7.2 with the dna_r10.4.1_e8.2_400bps_sup.cfg configuration, ensuring that only reads with quality score greater than 10 were included [9].

Host contamination as well as potential human DNA contamination were removed by mapping the obtained reads to *Manihot esculenta* V8 genome as well as the Human Genome GRCh38.p14 using Minimap2 version 2.28 [10,11] with the pre-set ‘map-ont’ parameters for nanopore data. Unmapped reads were then filtered using the appropriate bitwise flag (-f 4) in Samtools version 1.20 [12].

De novo assembly was conducted using Flye version 2.9.4 [13]. The assembled contigs were then classified against NCBI viral Refseq protein database (release 228) using Diamond version 2.1.10 [14]. Following taxonomic classification, three samples, each from a different leaf sampled in separate locations and years, returned hits matching Genomoviruses and specifically the *Gemycircularvirus* genus.

### 2.3. Sequencing of Full Genomovirus Sequences

The contigs matching Genomoviruses were used to design a pair of abutting primers (Gemy_F–5′-CGTTCCCTTCCAAGTCGGT-3′, Gemy_R–5′-TCATAAYGATGGCGGAACT-3′) to facilitate the full genome amplification using PCR. Amplification was conducted on the purified RCA product of the three samples using the NEB OneTaq polymerase kit with the following reaction conditions: initial denaturation at 98 °C for 2 min followed by 34 cycles of 98 °C for 30 s, 55 °C for 30 s, 68 °C for 90 s and then a final 68 °C for 10 min. The PCR amplicons were then resolved on 1% agarose gel. Successful PCR reactions were then sequenced on the nanopore platform at Plasmidisaurus (Eugene, OR, USA).

### 2.4. Bioinformatic Analyses

Assembly of the obtained reads was performed by one of two complementary approaches: using long-read de novo assembly software or generating a consensus through seed-based reference polishing. For de novo assembly, three long-read assemblers including Flye version 2.9.4 [13], Canu version 2.2 [15] and Raven version 1.8.3 [16] were employed using default parameters. Where an assembler successfully resulted in contigs, the obtained contigs were assessed for completeness, i.e., a length of approximately 2.2 kb. Contigs of appropriate length were then polished with the raw reads using two rounds of Racon version 1.5.0 [17] and one round of Medaka 1.11.3 (ONT).

Where none of the assemblers resulted in any usable contigs, mostly due to the lack of significant overlap given that the reads are the result of amplicon sequencing, consensus was generated using a seed-based reference polishing approach. In this approach, reads were first filtered to exclude reads longer than 2.2 kb and with quality lower than 15 using NanoFilt version 2.8.0 [18]. The filtered reads were then sorted by size to extract the longest read using SeqKit version 2.8.2 [19]. This read was then converted to FASTA and used as a seed for subsequent polishing rounds. The seed was polished by mapping high-quality raw reads to it using Minimap2 version 2.28 [10] followed by two rounds of Racon and one round of Medaka polishing. The final polished consensus sequences were then annotated in Geneious Prime 2025.

### 2.5. Genome Annotation and Motif Scan

The obtained genomes were annotated with the ORF finder in Geneious Prime 2025 using a Genomovirus Reference sequence (accession no. NC 076496) as a guide. Cressent version 1.0.0 [20] was used to find common rolling circle replication (RCR) motifs as well as predict stem loop structures while RNAfold version 2.4.7 was used to visualise the predicted stem loop structures.

### 2.6. Phylogenetic Analysis

The pairwise identity of sequences obtained in this study and existing genomovirus species was obtained using the Sequence Demarcation Tool (SDT v1.2). Rep and CP sequences were aligned with representative genomovirus sequences using MAFFT with default settings while maximum likelihood phylogenetic trees were constructed with IQ-TREE2 [21] using automatic model selection and 1000 ultrafast bootstrap replicates. The trees were visualised using FigTree version 1.4.4.

## 3. Results

### 3.1. Recovery and Sequencing of Full Genomovirus Genomes

Three Genomovirus isolates were recovered from leaves originally collected for the isolation of Cassava mosaic begomoviruses. The Genomoviruses were unexpectedly discovered during metagenomic analysis, successfully amplified using abutting primers and sequenced. Sequencing yielded between 601 and 2970 reads per sample with average read lengths ranging from 627 to 823 bp. Isolate b52 was successfully assembled using Flye while the other two isolates were assembled via seed-based polishing. The final assemblies ranged from 2090 to 2188 nucleotides in length with a GC content of between 47.3% and 47.7% (Table 1). BLASTn analysis revealed that the isolates had 84.81–95.37% similarity with isolates available in NCBI GenBank (Table 1). The complete genome sequences of the three isolates obtained in this study have been deposited in GenBank under accession numbers PX447322-PX447324 and are also available in Supplementary File S1.

### 3.2. Genome Characterisation

**Figure 1:**
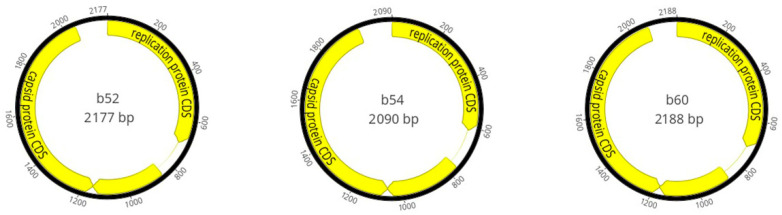
Genome map of Genomovirus isolates obtained in this study.

All three assembled genomes exhibited two open reading frames (ORFs), including the replication-associated protein (Rep) and capsid protein (CP) in an ambisense orientation (Figure 1). The Rep protein ranged from 305 to 340 amino acids in length while CPs ranged from 300 to 302 amino acids in length. Each genome contained one intergenic region between both ORFs which varied in length ranging from 116 to 129 nucleotides long. There was no evidence of a second intergenic region as the 3′ end of both ORFs were juxtaposed. Secondary structure prediction revealed the presence of stem loop structures with the conserved nonanucleotide motif TATTTATAA across all three isolates.

The Rep protein of all three isolates shared an overall pairwise identity of 89.3% with the highest similarity observed between isolates b52 and b60 at 92.4%. Comparing the rep genes to previously available sequences, b52 shared 97.6% amino acid similarity with a Gemycircularvirus isolated from nasal swabs (YP_010798590.1), while b54 and b60 shared 88.9% and 92.33% identity, respectively, with the same isolate. The coat protein, in contrast, showed significantly lower similarities with an overall pairwise identity of 62.1% between the isolates. Isolates b52 and b54 shared 100% pairwise amino acid similarity while b60 shared 43.7% pairwise similarity with b52 and b54. When comparing to previously available sequences, b52 and b54 shared 76.98% identity with a Gemycircularvirus isolated from nasal swabs (YP_010798589) while b60 shared 41.67% identity with a Genomovirus obtained from human serum metagenome (UGV24164.1).

### 3.3. Conserved Motifs

Analysis of motifs across all three isolates revealed the presence of conserved RCR and helicase motifs typically found in CRESS-DNA viruses. RCR I (LLTYSQ) and RCR III (YAGK) motifs were present and conserved across all three isolates. RCR II showed less conservation, with a variation in the last residue, i.e., valine (THFHV) in b52 and alanine (THFHA) in b60. The RCR II motif was, however, not detected in isolate b54 even though it was detected in accession NC_076496.1 which was the closest match in the GenBank database (Figure S1, Supplementary File S2). The GRS (gemini-like replication sequence) domain, which plays an essential role in DNA binding and cleavage, was detected in all three isolates, with b52 and b54 having an identical GRS sequence (FDVSDKYRHKKRMHWGK) while b60 showed some variation at the terminal region of the motif (FDVSDKYRHKKRLQWGK). The helicase-related motifs, Walker A (GKTRLGKT), Walker B (VFDDI) and Motif C (WEEN) were conserved across the three isolates with no amino acid substitutions observed.

### 3.4. Phylogenetic Inference

**Figure 2:**
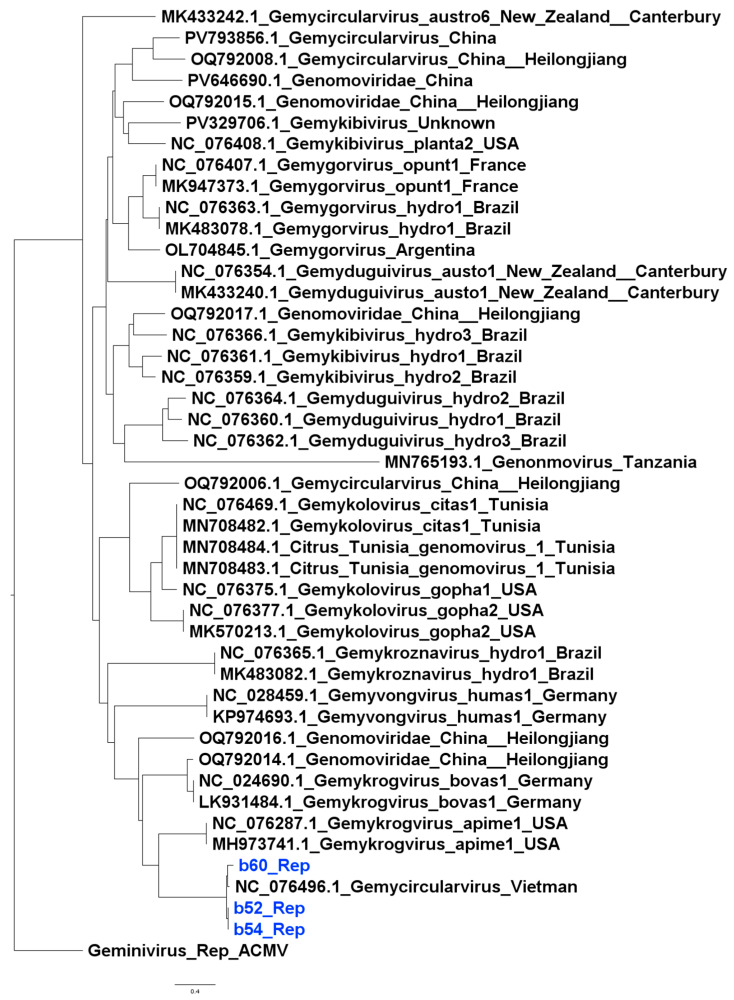
Maximum likelihood phylogenetic tree based on Rep nucleotide sequences with Geminivirus Rep as the root. Isolates from this study are shown in blue.

**Figure 3:**
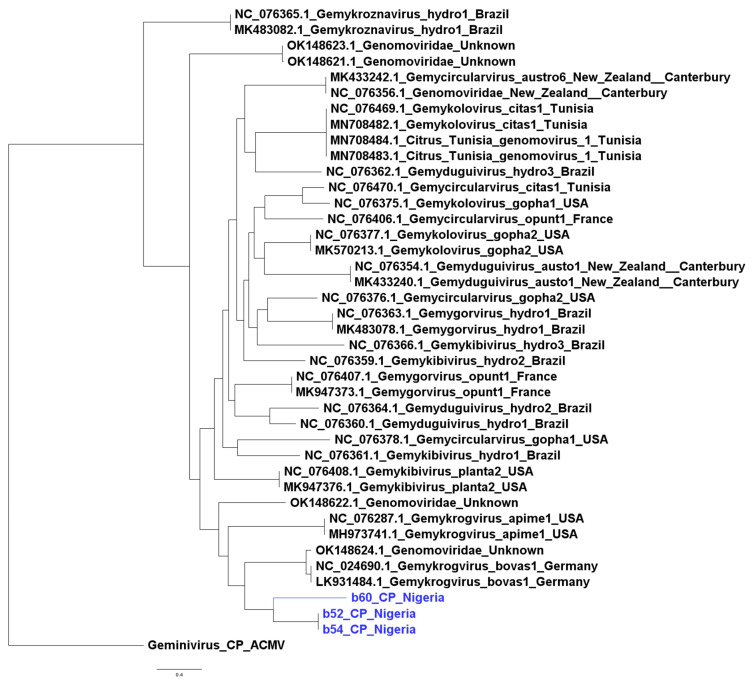
Maximum likelihood phylogenetic tree based on capsid protein nucleotide sequences with Geminivirus capsid protein as the root. Isolates from this study are shown in blue.

The isolates from this study clustered with the wider Genomovirdae family; however, they did not cluster into any well-defined genus based on rep nucleotide sequences. They formed a cluster alongside one previously isolated Genomovirus from Vietnam, suggesting that they may represent a divergent lineage within the family (Figure 2). Similar patterns were observed with capsid protein sequences (Figure 3).

## 4. Discussion

The Genomoviruses described in this study add to the growing body of evidence on the distribution and diversity of members of the Genomovirus family. While the sequences obtained in this study share important similarities with other members of the family, they also have significant differences at the nucleotide and amino acid levels, hinting at a divergent lineage within the family. However, based on the current threshold by the ICTV, the isolates from this study most closely resemble isolates belonging to the *Gemycircularvirus* genera. This taxonomic ambiguity is typical among members of the family and represents a broader challenge with Genomovirus systematics where newly discovered sequences often share conserved architectures but do not often form tightly clustered groups with existing genera [1,22,23].

Beyond the taxonomic implications of the study’s findings, the ecological significance of the identified strains is unclear. While previous studies have found genomovirus species associated with several plant families [24], it is possible that their presence on the cassava leaves reflects environmental contamination introduced through insect vectors, as opposed to an actual infection of the plant [25]. The most plausible explanation, however, is that the viruses are part of the virome of other organisms, such as fungi, colonising the cassava leaves. This is consistent with current knowledge of the host range of genomoviruses [26,27]. Determining the exact role of the Genomovirus in the cassava leaves would require targeted host association studies which are beyond the scope of the current study.

An important finding from this study was the absence of the RCR II motif from one isolate. This motif is largely conserved across the Rep protein of CRESS DNA viruses and plays a crucial role in initiating rolling circle replication [22]. The absence of this motif could be indicative of a divergent replication mechanism or failure to detect the motif due to sequence variability, especially since motif discovery focused on known conserved Genomovirus motifs, or perhaps a true loss of function compensated by structural changes to the Rep protein. It is important to note that such changes are rarely reported in the literature however a previous study focusing on metagenomic retrieval of full genomes also reported missing or partial RCR II motifs [28]. Overall, the findings from this study reinforce the genetic plasticity found within the Genomovirus family.

## 5. Conclusions

The study adds to the growing body of evidence on CRESS-DNA viruses and especially Genomovirus diversity. While the isolates in this study shared several important similarities with members of the family, their phylogenetic placement suggests the existence of a divergent lineage underscoring taxonomic gaps in the understanding of this viral family.

## Figures and Tables

**Table 1:** Summary of identified Genomoviruses.

Isolate	Year/Location	No Reads	Coverage	Min Base Coverage	Genome Length (bp)	GC (%)	BLASTn Search Results			
							Best Hit	% Coverage	E Value	Identify
b52	2015/Benue State	2557	779.81×	228x	2177	47.50	MN765193.1	100%	0.0	95.37%
b54	2015/Oyo State	601	162.17×	120x	2090	47.61	NC_076496.1	95%	0.0	84.81%
b60	2017/Ondo State	2970	1015.83×	23x	2188	47.76	NC_076496.1	57%	0.0	93.78%

Min base coverage—minimum base coverage; coverage—mean sequencing depth.

## Data Availability

All data and code used in this article can be found at the following GitHub repository https://github.com/bodeoni/Genomovirus_paper (accessed on 8 October 2025) Final polished sequences of the three isolates obtained in this study are available in NCBI GenBank Accession PX447322-PX447324. The sequences are also available in Supplementary File S1.

## Supplementary Materials

The following supporting information can be downloaded at: https://www.mdpi.com/article/10.3390/v17111418/s1, File S1: Sequences obtained from the study in Genbank format; File S2: Figure S1: Multiple sequence alignment of the three isolates from this study, Figure S2: Coverage Plot for all three isolates.
