# Supplementary material for: Identification and Full-Genome Characterisation of Genomoviruses in Cassava Leaves Infected with Cassava Mosaic Disease
Source: Viruses. 2025 Oct 25;17(11):1418. doi: 10.3390/v17111418 (PMC12656945; doi:10.3390/v17111418)
Supplement: Supplementary file 1 [file viruses-17-01418-s001.zip › File S2.pdf]

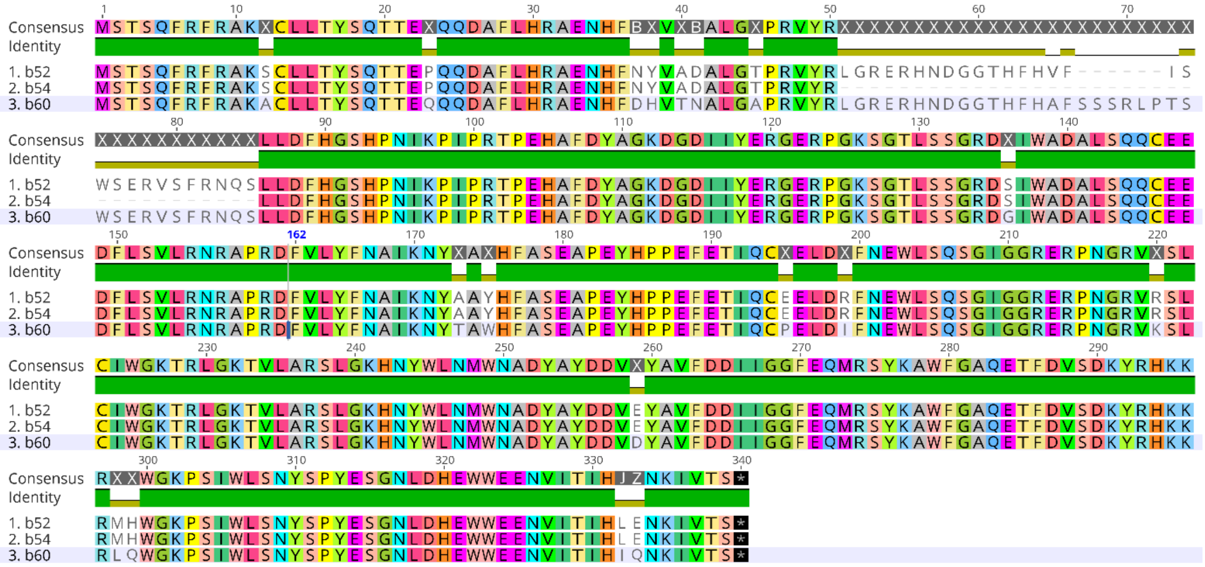

Figure S1: Multiple sequence alignment of the three isolates from this study

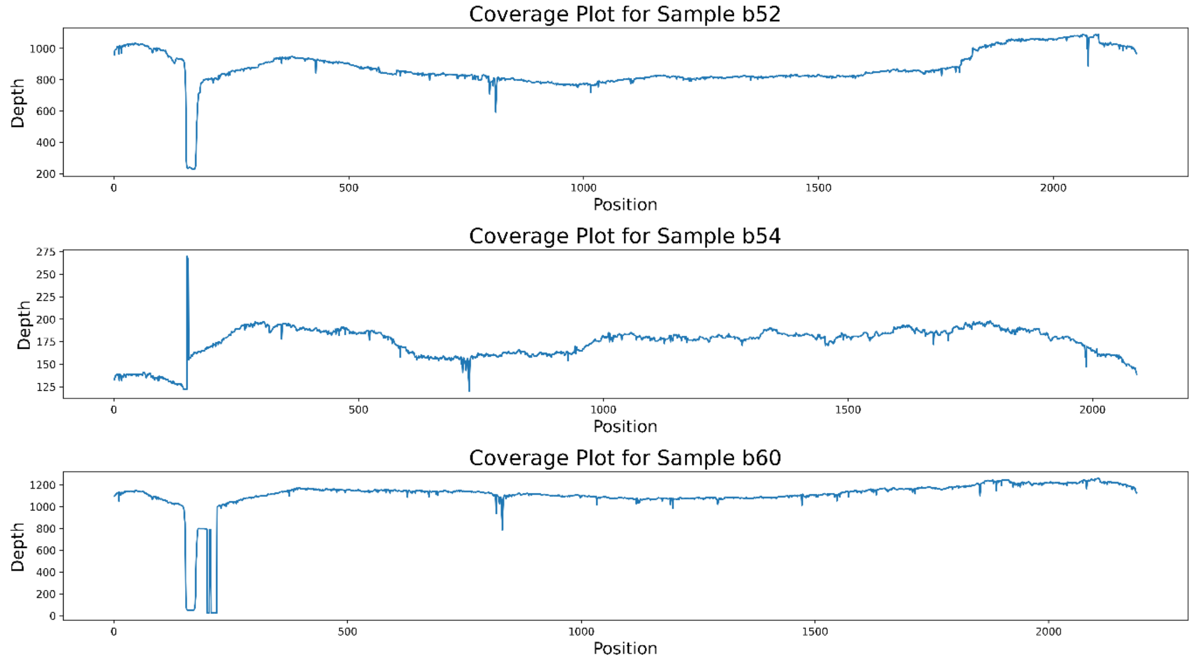

Figure S2: Coverage Plot for all three isolates
